# Activation of an inflammatory response is context-dependent during early development of the California sea lion

**DOI:** 10.1098/rsos.150108

**Published:** 2015-04-29

**Authors:** Camila Vera-Massieu, Patrick M. Brock, Carlos Godínez-Reyes, Karina Acevedo-Whitehouse

**Affiliations:** 1 Unit for Basic and Applied Microbiology, School of Natural Sciences, Autonomous University of Queretaro, Avenida de las Ciencias S/N, Queretaro 76230, México; 2 Institute of Biodiversity, Animal Health and Comparative Medicine, College of Medical, Veterinary and Life Sciences, University of Glasgow, Glasgow G12 8QQ, UK; 3 Cabo Pulmo National Park, Comisión Nacional de Áreas Naturales Protegidas, SEMARNAT, La Ribera, BCS, Mexico; 4 The Marine Mammal Center, 2000 Bunker Road, Sausalito, CA 94965, USA

**Keywords:** ecoimmunology, immune ontogeny, inflammation, phytohaemaglutinin, trade-off

## Abstract

Variations in immune function can arise owing to trade-offs, that is, the allocation of limited resources among costly competing physiological functions. Nevertheless, there is little information regarding the ontogeny of the immune system within an ecological context, and it is still unknown whether development affects the way in which resources are allocated to different immune effectors. We investigated changes in the inflammatory response during early development of the California sea lion (*Zalophus californianus*) and examined its association with body condition, as a proxy for the availability of energetic resources. We found that the relationship between inflammation and body condition varied according to developmental stage and circulating levels of leucocyte populations, a proxy for current infection. Body condition was related to the magnitude of the inflammatory response during two of the three developmental periods assessed, allowing for the possibility that the availability of pup energetic reserves can limit immune function. For older pups, the ability to mount an inflammatory response was related to their circulating levels of neutrophils and the neutrophil to lymphocyte ratio, implying that the infection status of an individual will influence its ability to respond to a new challenge. Our results suggest that trade-offs may occur within the immune system and highlight the importance of taking into account ontogeny in ecoimmunological studies.

## 2. Background

In natural populations, individuals vary greatly in the strength, specificity and efficiency of their immune responses [1]. It has been stated that these variations reflect differential allocations of energetic resources among costly physiological functions such as growth, development, reproduction and immunity [2]. Because resources are limited and their demands change through time, investing in a particular physiological function can decrease or restrict resource availability for other functions [3]. The costs of maintaining and deploying immune effectors can manifest differently through time, but are generally reported as negative correlations between aspects of immunity and other physiologically relevant traits, such as those related to reproductive success and growth [4–7].

In the past decades, scientists have attempted to disentangle the ways in which immune function varies in natural populations and have aimed to describe how allocating resources towards immunity impacts other physiological functions [8]. For example, from studies conducted in birds, mammals and reptiles, it is known that immune function can decrease during the reproductive season [9], and an increase in immune function can decrease reproduction [10]. Resource allocation has been hypothesized to depend on communication between the neuroendocrine and immune systems [11], and body condition is thought to be a suitable proxy for resource availability due to such communication. For instance, mammalian white adipose tissue helps support costly functions, including immunity [11–14], via endocrine fatty-tissue effectors such as leptin, a hormone with known immune effects [15–17]. To date, although the mechanisms by which immune-derived costs are implemented are not completely clear, and we do not fully understand how these costs contribute towards maintaining immune system heterogeneity in natural populations [1], availability of energetic resources appears to be fundamental in defining the nature and magnitude of various immune responses among individuals.

Interestingly, despite the growing interest in understanding the costs of deploying or maintaining immune effectors in natural populations, rarely has ontogeny been taken into account in these studies. This is unfortunate, as there is evidence that not only does the immune system develop differently among taxonomic groups, but also that at an individual level, the ability to respond to immune challenges differs through time [18]. For example, *Drosophila melanogaster* larvae exposed to parasitoid wasps (*Asobara tabida*) decreased their feeding rates and were less likely to survive than adult flies similarly challenged [19,20]. Furthermore, the activation of specific immune responses during early development can alter other physiological traits during adulthood [21]. Collectively, these studies suggest that the rules which govern resource allocation will vary among developmental stages. The need to bridge the gap between cost-focused studies and developmental studies has already been identified by ecoimmunologists [2], as such studies could help reconcile whether variations in immune function are owing to differences in resource allocation or are a consequence of immune development *per se*, and in the long run, they could help us understand how early life-history experiences may affect immune traits during adulthood.

We aimed to examine variation in innate immune deployment during early mammalian development and address questions regarding the potential costs of immune challenges. Using the California sea lion (*Zalophus californianus*) as a study model, we analysed the relationship between the inflammatory response to an exogenous mitogen and body condition, as a proxy for individual resource-availability, at distinct developmental stages. Different aspects of the immune system of the California sea lion have been addressed in the past decades, and taken together, these studies suggest that at the cellular and molecular levels their immune effectors are mostly similar to those observed in terrestrial carnivores [22], making their study feasible. Furthermore, studies conducted in other pinniped species have shown that pups have competent immune systems, but different immune effectors change as pups develop. For instance, in the Steller sea lion, *Eumetopias jubatus*, an otariid with a life history similar to the California sea lion, total leucocyte and neutrophil counts, as well as neutrophil to lymphocyte ratios (NLRs), decrease as pups age [23], a finding also reported for habour seals, *Phoca vitulina* [24].

Inflammation is one of the main innate immune responses and it represents the first line of defence against foreign agents [25]. As activation of an inflammatory response gives way to the recruitment of immune cells and humoural effectors to the damaged site [26], this process presumably requires increased investment of resources. Phytohaemaglutinin (PHA) has been used extensively as a mitogen to stimulate inflammation *in vivo* [26,27]. Response to PHA has been measured in other otariid pinnipeds [28,29], and the recorded inflammatory responses have been comparable to what is observed in other taxonomic groups. Most importantly, the cost of responding to PHA has been shown to be equivalent to the energy required for other essential physiological functions (see [30]). Thus, we tested the hypothesis that individuals with better body condition would be able to invest more resources in mounting an inflammatory response and that this relationship would vary between developmental stages.

## 3. Material and methods

### 3.1 Sample collection

All fieldwork was conducted in the California sea lion breeding colony of Granito Island (29°33′43′′ N, 113°32′04′′ W), in the Gulf of California. Sea lion pups were captured with the use of hoop nets and were restrained manually during sampling. We collected samples from pups at three distinct periods of their development; 11 pups of six to eight weeks of age were sampled during July 2012, 18 pups of 22–24 weeks of age were sampled during October 2012 and 23 neonate pups (one to three weeks of age) were sampled during June 2013. Each cohort consisted of pups that had not been sampled in the previous sampling trip in order to avoid any error associated with stronger immune responses to a secondary PHA challenge [27].

All pups were marked by shaving a number tag in their dorsum, and by one of the following methods: (i) insertion of an Avid microchip (pups sampled in July 2012 and June 2013), or (ii) flipper tags with a unique identification number on both anterior flippers (pups sampled in October 2012). Each pup was weighed using a vertical hanging scale (max. 60 kg; 0.1 kg precision) and measured to obtain standard length. Visual inspection by a veterinarian ascertained the presence of ectoparasites and lesions, as well as a general health index for each pup. We collected 5–7 ml of blood from the caudal glutaeal vein of each pup in a Vacutainer tube containing EDTA as a preservative.

### 3.2 Immune challenge

Immediately following blood collection, each pup was challenged with PHA, a lectin derived from red-kidney beans and commonly used for ecoimmunology studies [27]. PHA is thought to both stimulate T lymphocyte mitogenesis and cause local inflammation as a direct result of tissue damage, with the type of effect dependant on the presence of specific cell-surface receptors to bind PHA and the time elapsed since exposure [31]. To conduct the PHA challenges, we first measured the thickness of the webbing between the second and third digits of both hind flippers. Next, we injected 100 μl of 1 mg ml^−1^ PHA solution intradermally into the right flipper, and 100 μl of sterile saline solution into the left flipper. This was done to account for any inflammation caused by mechanical irritation of the injection *per se* and was considered as the control for each challenge. As we had previously determined that there were no differences between the magnitude of the PHA-induced inflammation observed at 4 h and that which was observed at 24 h post-injection (see the electronic supplementary material, figure S1), in order to avoid recapture and minimize stress, we maintained the pups in a shaded pool during 4 h following the PHA challenge. Inflammation was assessed after this time by re-measuring webbing thickness in both hind flippers. All measurements were taken in triplicate to the nearest 0.01 mm using a thickness gauge (Mitutoyo, USA). Pup response to PHA was calculated as the difference between the saline-induced change in median thickness of the left flipper and the PHA-induced change in median thickness of the right flipper [30].

### 3.3 Histology

In order to correctly interpret the results of a PHA challenge, it is necessary to characterize the inflammation histologically [32]. For this, after measuring both flippers, biopsies were taken from the centre of the PHA- and saline-injected tissues with a 2 mm diameter tissue corer (Biopsypunch, Miltex, USA) for two-month-old pups, and with a 4 mm diameter tissue corer (Biopsypunch, Miltex) for six-month-old pups. After sampling, haemostatic pressure was applied to the wound and the site was liberally sprayed with antiseptic betadine. Biopsies were not taken from neonate pups owing to permit restrictions. All pups were released following the biopsy. The biopsies were immediately fixed in 10% buffered formalin and protected from sunlight.

Tissue samples were dehydrated with the use of a histokinnette (Microm STP 120), embedded in paraffin, sectioned, mounted on slides and stained with haematoxylin and eosin following standard procedures (see the electronic supplementary material). Cell infiltration was quantified in PHA- and saline-injected tissues by scanning two slides at 40× on an Aperio ScanScope CS (Aperio Vista, CA, USA) and visualizing the images in ImageJ (National Institute of health, Bethesda, MD, USA). We generated a rectangular box of fixed-width (120 μm) and counted all the immune cells inside this area, including those cells in contact with the perimeter of the box. For each individual pup, three counting areas in each PHA- and saline-injected tissue sections were defined for cell quantification, and the averages of these three subsamples were used for statistical analysis.

We quantified neutrophils, lymphocytes, eosinophils, basophils, macrophages and fibroblasts in the above-mentioned counting area. Lymphocytes were identified as circular cells with large, purple nuclei that filled almost the entire cell. Neutrophils were identified by their multi-lobed nuclei, eosinophils by their reddish cytoplasmic granules, basophils were characterized by the presence of dark basophilic granules and macrophages were identified by their large size and kidney-shaped nucleus [33]. Finally, fibroblasts were identified by their elliptic and enlarged nucleus [34].

### 3.4 Haematology

Following collection, blood samples were kept in a cooler until processed in the field camp (between 8 and 12 h following collection). We performed total leucocyte counts using a haemocytometer (Optic Labor, CA, USA) after diluting 50 μl of blood in 950 μl Turk solution (crystal violet, and 6% acetic acid). We prepared three blood smears per sample and fixed them in 90% methanol before staining with buffered Wright solution. White blood cell differentials were reported for neutrophils, lymphocytes, monocytes, eosinophils and basophils. Differentials were reported as percentages of each leucocyte type out of 100 cells in each blood smear. To calculate absolute numbers for each leucocyte type, the total white blood cell count of each case was multiplied by the percentages of the different leucocyte types. Basophils were excluded because they were rarely observed [22]. NLR was calculated for each pup as a marker of subclinical inflammation [35]. The haematocrit and plasma protein (optical density, using a densitometer) were determined for each sample.

### 3.5 Measuring body condition

We estimated body condition for each pup by calculating mass per unit length. For this we obtained the scaled mass index (SM_*i*_) proposed by Peig & Green [36]. The relationship between body mass and length has been used as a measure of condition for many vertebrates [29,37]. Specifically, the SM_*i*_ takes into account the scaling between body components and body size. This is important because energy stores are related to structural size in a complex manner [36].

### 3.6 Statistical analysis

The distributions of infiltrated cell type counts per age class were examined visually and by performing a Shapiro–Wilk normality test [38]. Variables whose distributions differed from the normal distribution were eosinophil counts in two-month-old pups and macrophage and eosinophil counts in six-month-old pups. Differences in cell infiltration and swelling in PHA- and saline-challenged tissues were examined using independent sample *t*-tests for variables with normal distribution and with Mann–Whitney–Wilcoxon tests for variables that were non-normally distributed [38].

To test the effect of body condition and blood parameters on skin-fold thickness in response to the PHA challenge, we constructed generalized linear models (GLMs). Owing to missing blood data for some of the two-month-old pup cohort and to the non-normal distribution of data for the six-month-old cohort, we analysed each sampling period separately. For the two-week-old pup cohort, we fitted two Gaussian GLMs; in the first model, we defined change in skin-fold thickness as the response variable and body condition, number of neutrophils and total leucocytes as explanatory variables. In the second model, we defined change in skin-fold thickness as the response variable and body condition, NLR and total leucocytes as explanatory variables. The model included third-degree interactions between variables. For the two-month-old pups, we fitted a Gaussian GLM where we defined change in skin-fold thickness as the response variable and body condition as the explanatory variable. Blood parameters were not included in the model owing to incomplete data. Finally, for the six-month-old pups, as response to PHA was dichotomous (i.e. either it occurred or it did not), we fitted three binomial GLMs; in the first, we defined change in skin-fold thickness as the response variable, and body condition and neutrophil count as explanatory variables. In the second, we defined change in skin-fold thickness as the response variable and body condition and NLR as explanatory variables. In the third, we defined change in skin-fold thickness as the response variable and body condition and total circulating leucocytes as explanatory variables. Models were constructed independently for this age class to avoid over-dispersion and lack of power due to relatively small sample sizes [38]. Neutrophils and NLR were included as explanatory variables because they reflect the most abundant leucocyte populations in California sea lion blood and are considered diagnostic for clinical assessment of health [22]. All models were checked for over-dispersion, and their best fit estimated by comparing their Akaike information criteria (AIC). All analyses were performed in R v. 2.14.1.

## 4. Results

### 4.1 Histological assessment of inflammation

**Figure 1.**
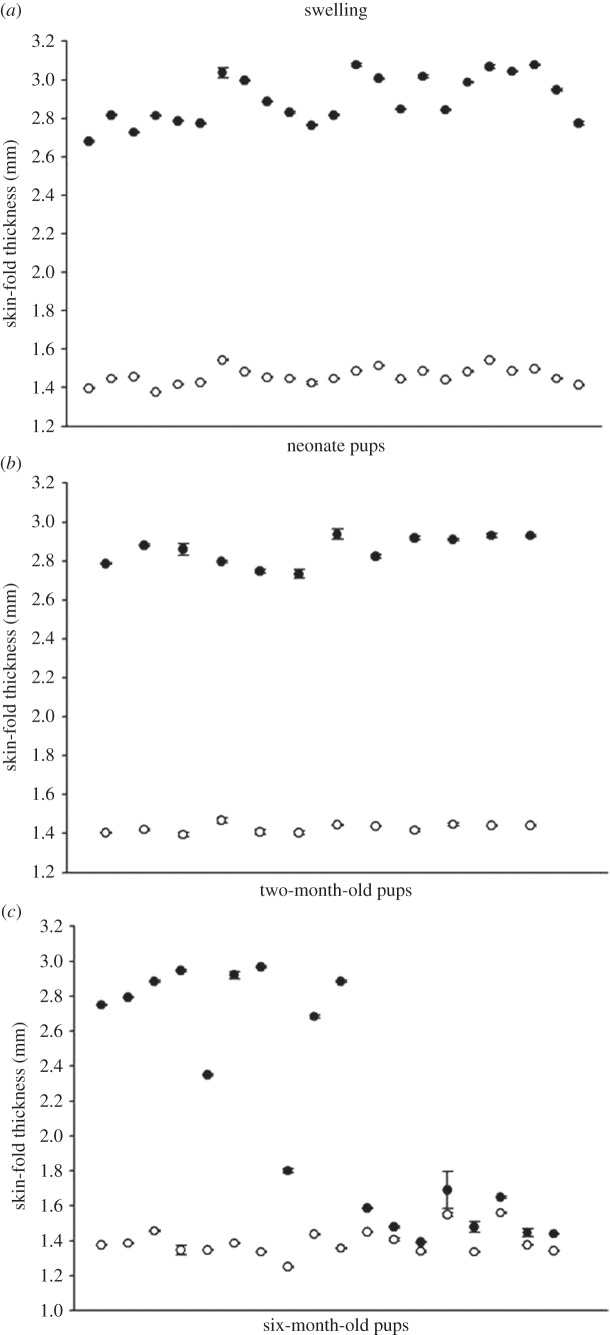
Comparison of swelling in PHA-injected (closed circles) and saline-injected (open circles) flipper webs of (*a*) neonate, (*b*) two-month-old, and (*c*) six-month-old California sea lion pups. Graphs show individual data points (raw data) for the median of the three flipper measurements for each pup ±1 s.e.

All neonate pups showed significant swelling following the PHA challenge (two-tailed *t*-test, *t*_22_=50, *p*<0.0001; figure 1*a*) and a similar pattern was seen for two-month old pups (two-tailed *t*-test, *t*_10_=48.43, *p*<0.0001; figure 1*b*). By contrast, six-month-old pups had a dichotomous response to PHA; while some were unable to respond to the challenge (*W*=47.5, *n*=9, *p*>0.05; figure 1*c*), others showed significant swelling (*W*=81, *n*=9, *p*<0.01; figure 1*c*). Regardless of age, all skin biopsies from the PHA-challenged site of pups that showed swelling had a significant infiltration of leucocytes compared with the paired skin biopsy control (see the electronic supplementary material, figure S2). Neutrophils (average=26.17±1.08 s.d.) and lymphocytes (average=8.85±0.64 s.d.) were the most common leucocyte types in the PHA-challenged tissue, with few macrophages (average=5.35±0.74 s.d.) and eosinophils (average=1.38±0.28 s.d.) observed. Six-month-old pups that did not respond to PHA did not show infiltration of any cell type and the tissue resembled the skin biopsy control (see the electronic supplementary material, figure S1).

### 4.2 Inflammation, body condition and age

**Figure 2.**
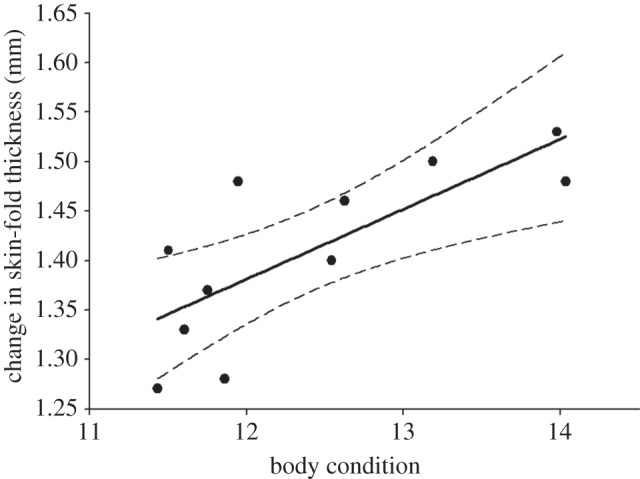
Relationship between change in PHA-induced skin-fold thickness and body condition of two-month-old California sea lion pups. Adjusted *r*^2^=0.53, *p*=0.006. Dotted lines represent 95% CIs.

Pup body condition varied between sampling stages. Specifically, two-month-old pups had lower body condition than neonates and six-month-old pups (*F*_2,51_=3.71, *p*=0.034; see the electronic supplementary material, figure S2). For neonates, the change in skin-fold thickness following the PHA challenge was not related to body condition, circulating neutrophils, NLR, total white blood cells, or to their interactions (electronic supplementary material, table S1). However, for two-month-old pups, PHA-induced inflammation was linearly related to body condition (figure 2, adjusted *r*^2^=0.53, *p*=0.006).

**Figure 3.**
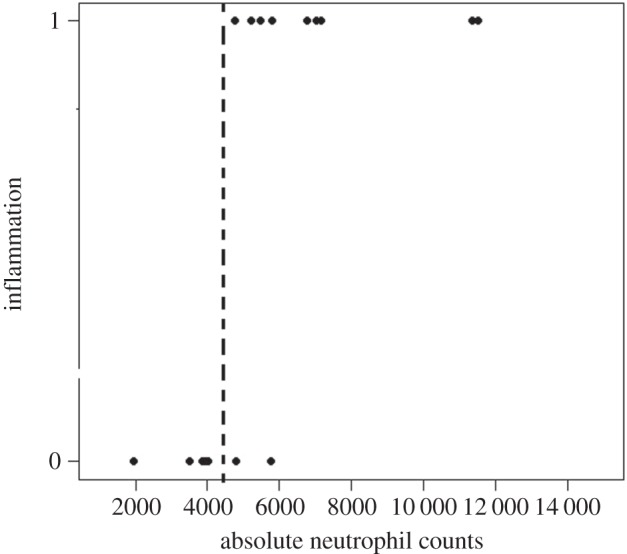
Presence or absence of swelling in terms of numbers of circulating neutrophils per microlitre of blood of six-month-old California sea lion pups.

**Figure 4.**
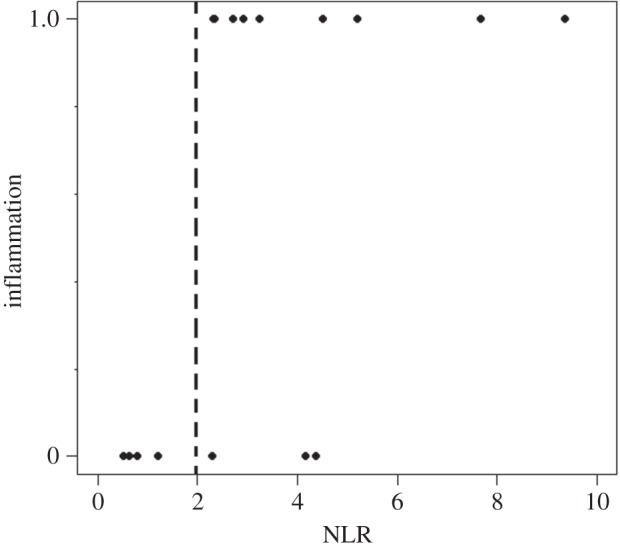
Presence or absence of swelling in terms of the NLR of six-month-old California sea lion pups.

As mentioned above, the PHA swelling response in six-month-old pups was dichotomous, and thus did not follow a normal distribution. According to the observed histology, we established that a change in skin-fold thickness below 0.7 mm was equal to no swelling because there was no leucocyte infiltration in these tissues; therefore, we transformed the data to binary form. By fitting a binomial GLM, we observed that inflammation was not related to body condition when examined alone (*χ*^2^_17_=0.002, *p*=0.9868). Nonetheless, the response to PHA was positively correlated to the number of circulating neutrophils (*p*=0.0002; see model (A) in table 1; figure 3). The NLR also explained a pup's response to PHA, as animals with relatively lower NLR (average=2.00±1.66 s.d.) were unable to mount a response than pups with a relatively higher NLR (average=4.47±2.52 s.d.; see model (B) in table 1; figure 4). We observed interesting interactions that also helped explained the six-month-old pups' ability to respond to PHA. Specifically, the NLR and its interaction with body condition explained a pup's ability to mount an inflammatory response when challenged (*p*=0.012, see model (B) in table 1). Also, when considered in interaction with the total number of leucocytes, body condition helped explain the ability to respond to PHA (*p*=0.011, see model (C) in table 1).

**Table 1.** Changes in skin-fold thickness (PHA-induced swelling) in six-month-old California sea lion pups as an effect of changes in: (A) body condition (SM_*i*_), numbers of circulating neutrophils per microlitre of blood (NEU); (B) neutrophil to lymphocyte ratio (NLR); and (C) total white blood cells per microlitre of blood (WBC). (Table shows the output of the GLMs, including significant interactions.)

variable	estimate	*p*(>*χ*)
model (A): swelling∼SM_*i*_×NEU, family=binomial		
SM_*i*_	−4.331	0.565
NEU	13.31	0.0012
SM_*i*_ : NEU	−1.407	0.235
*n*=18, d.f.=17, AIC=15.11		
model (B): swelling ∼ SM_*i*_×NLR, family=binomial		
SM_*i*_	−2.984	0.747
NLR	13.722	0.014
SM_*i*_ : NLR	−1.1856	0.012
*n*=18, d.f.=17, AIC=17.56		
model (C): swelling ∼ SM_*i*_×WBC, family=binomial		
SM_*i*_	−10.477	0.565
WBC	0.0196	0.781
SM_*i*_ : WBC	−0.0015	0.001
*n*=18, d.f.=17, AIC=19.73		

## 5. Discussion

In this study, we have shown that the inflammatory response induced by PHA involves multiple leucocyte populations, with no specific cell type being exclusively responsible for the effect. In this sense, our study provides evidence that the PHA challenge can be considered a valid measure of non-specific pro-inflammatory capacity.

In order to survive and reproduce, individuals must distribute resources among competing physiological processes in a way that complements current, often changing, environmental conditions [39,40]. In this study, we tested whether availability of energetic resources, assessed indirectly as body condition, during different developmental stages of a carnivore marine mammal was relevant to mounting an inflammatory response. We also assessed if infection status, as inferred from circulating leucocyte populations, impinged on inflammation. The inflammatory response is one of the most important non-specific mechanisms of a vertebrate's immunity, and as such is likely to be a costly physiological function that can be constrained by resource availability [41,42]. In this sense, we predicted that body condition as a measure of resource availability would be positively related to the occurrence and magnitude of induced inflammation, and that such a relationship would be maintained through development. Nevertheless, we found that the relationship between body condition and inflammation appeared to be context-dependent, as it varied according to the developmental stage of the pups.

Contrary to what we predicted, neither body condition nor circulating numbers of leucocyte types were related to the magnitude of the inflammatory response of neonate pups. Immunological costs can be masked by the underlying environmental conditions [43,44] and immunological costs can sometimes be evident only during times of diminished resource and nutrient availability [45,46] or anthropogenic stress [29]. In this sense, it is possible that the neonates of our study have not yet experienced immune challenges that require considerable energetic investment. This possibility is supported by the fact that all neonate pups were in good body condition. It is also possible that our results point towards endocrine-immune associations. The endocrine system is known to be an important regulator of physiological trade-offs [47] and it has been proposed that the hormone leptin modulates trade-offs with the immune system [48]. Leptin is known to have multiple immunostimulatory effects [48,49] and there is evidence in humans [50] and rats [51] that this hormone is transferred from mother to offspring via the milk during lactation. Leptin concentrations are known to be higher in the colostrum and during the first stages of lactation and they plateau as offspring grow [52,53], therefore it is possible that high concentrations of leptin at two weeks of age are affecting the way in which neonate pups respond to immune challenges (see [54]).

It is interesting to note that we found body condition of two-month-old pups was roughly 10% lower than that of neonate and six-month-old pups. This unexpected finding suggests that between birth and two months of age, pups might have experienced one or more events that impacted negatively on their condition. In the Channel Island rookeries, it is common for young California sea lion pups to be infected by the haematophagous hookworm *Uncinaria* spp. [55] that can cause anaemia, enteritis and peritonitis [56]. Intensity of infection is particularly high in two-month-old pups, and numbers of hookworms decrease to minimum levels at around seven months of age [55]. However, hookworm infections have not been reported for California sea lions within the Gulf of California, and no hookworms have been detected during necropsies nor following molecular analysis of faeces in pups from Granito rookery (K.A.-W. 2014, personal communication), where the present study was conducted. Thus, it is unlikely that hookworm infections could explain the observed pattern. On the other hand, it is known that California sea lion pups increase their maintenance requirements between the first and second months of life [57]. Such an increase in maintenance requirements could explain the observed changes in pup body condition. Moreover, in line with our prediction, two-month-old pups had a positive relationship between body condition and inflammation. As such, this pattern was not observed for the other age classes studied, although six-month-old pups showed an interesting interaction between body condition and NLR. A recent study conducted on Galapagos sea lions reported a similar association between condition and various immune parameters, but only for a colony that was not considered to have anthropogenic impact [29]. Based on our results we suggest that the possibility of investing resources to deploy an immune response will depend on the condition of an individual, but only in specific contexts. In other words, sea lions will invest in immunity and growth according to the resources available to them; those individuals that have access to more resources may invest more in both immunity and condition, a situation which would be expected under the hypothesis of phenotypic correlation (see [29,58]).

For six-month-old pups, we observed an interesting and rather unexpected pattern. Not all animals of this age class were able to respond to PHA. This phenomenon had not been shown previously and supports the idea that PHA-induced immune activity is costly [5]. In these pups, the ability to mount a response was directly related to their infection status, inferred by the circulating levels of leucocyte populations (see [59,60]). Pups that were unable to respond to PHA had fewer than 4800 neutrophils μl^−1^ (less than 55% of the total circulating leucocytes). Veterinary medical literature indicates that a percentage of neutrophils below 50% is out of the normal (healthy) range for California sea lion pups [22,60], suggesting that the health of pups that did not respond to PHA was compromised. Likewise, we observed a negative relationship between inflammation and the NLR. Here, the permissive threshold for inflammation was an NLR of 2.0, which is also in agreement with the clinical parameters reported for healthy California sea lion pups, where the expected proportion of neutrophils to lymphocytes is roughly 2.3 [22,60]. Lower NLR values imply that the proportion of lymphocytes is higher than expected, a finding that usually implies a severe bacterial- or acute viral infection [22]. In this sense, our results suggest that the health status of an individual at the time it encounters a new immune challenge will determine its ability to respond to the secondary challenge. Nevertheless, body condition will still play an important role determining such interactions, as pups whose neutrophils and lymphocytes were within normal ranges, but had a relatively poor condition, were unable to mount an inflammatory response.

Our results highlight the possibility that trade-offs also occur within the immune system itself. Even though this possibility still remains relatively unexplored in ecoimmunological studies, there is some evidence that such a situation occurs. For example, it has been shown that the occurrence of simultaneous immune challenges can negatively affect the progression of one of them [61]. This could be owing to the high cost of mounting immune responses, or it could reflect the effort of preventing anaphylaxis, septic shock or activation of costly febrile responses [59,61]. Likewise, another study showed that deer mice (*Peromyscus maniculatus*) infected with Sin Nombre virus had a lower swelling response when challenged with PHA [62]. The interaction between body condition and health status as determinant factors for mounting an inflammatory response suggests that for apparently ill six-month-old California sea lions it is too costly to invest energetic resources to face a second immune challenge.

The frequency and intensity with which an organism is exposed to pathogens within its environment can affect the magnitude and nature of the immune responses and change the organization of its effectors [4,58]. For example, in humans, absence of exposure to pathogens during the first year of life is associated with an elevated production of IgE and TH2-type cytokines [63]. It is also well known that pathogen exposure affects growth rate negatively in chickens [64] and pigs [65]. There is evidence suggesting such effects occur in the Galapagos sea lion, in which exposure to a rich pathogenic environment appears to generate a heightened immune response that impacts negatively on body condition [66]. Interestingly, weaning begins at six months of age for the California sea lion, leading to diet change as pups gradually include fish in their diet [67]. This process increases chances of exposure to a larger diversity of pathogens. The dietary transition that occurs during weaning is accompanied by profound modifications of intestinal physiology including marked cellular proliferation and differentiation [67,68], a process well described for rodents. While such occurrences have not been described for the California sea lion, it is likely that a similar change takes place. Therefore, if we envisage a scenario marked by high energetic demands necessary to support cellular process (differentiation and growth) and an increase in pathogen exposure induced by dietary changes, it is possible that our results show some immune functions of the weaning California sea lion pups are being compromised if their resources are used for other physiological processes.

One of the most important findings of our study was that ontogeny can affect allocation of energetic resources between different processes and, therefore, will impact the way in which immune-deployment costs are expressed. We showed that body condition, as a measure of resource availability, affects the occurrence and magnitude of PHA-induced swelling in different ways according to the developmental stage of the pups. It is known that immune function of an organism is dependent on its context. In this sense, the pattern we observed could be owing to environmental changes that affected, for example, maternal access to prey [69]. Nevertheless, body condition of six-month-old pups was not different from that of neonate pups, suggesting that, at least for the former, the patterns we observed are due to an ontogenetic effect. Evidence in support of this hypothesis is given by the report that, during weaning, several physiological changes occur that require substantial energetic input [70]. Furthermore, in pigs and humans, weaning has been reported to impact growth negatively [71,72] and it even has been compared to amphibian metamorphosis, as it is a very vulnerable period [68].

In comparison to other life stages, energetic demands during postnatal development are increased owing to growth maximization. Therefore, energetic trade-offs during this stage could be expected to be quite severe, particularly when resources are limited [57]. From a more applied viewpoint, perhaps one of the most important findings of our study was the inability to mount an inflammatory response following PHA challenge that we observed in some of the six-month-old pups. This is particularly relevant because the inflammatory response represents an organism's first line of defence against pathogens and absence of inflammation in infected or damaged tissue promotes bacterial colonization and subsequent infection [73]. It would seem that at six months of age, California sea lions are likely to be more susceptible to infection given the physiological processes they undergo during weaning. Species management plans and health-monitoring programmes could take our findings into account and focus their efforts on this particularly vulnerable period. Two-month-old pups also appeared to be vulnerable, but only if their body condition was poor, as their response to PHA was significantly lower than pups with good body condition. While a strong inflammatory response is not necessarily optimal, it is known that diminished inflammatory responses are associated with decreased survival [74]. Therefore, it seems worthy to consider two months of age as a vulnerable stage in sea lion development, especially if resource availability decreases, as happens during events such as El Niño-Southern Oscillation [75].

Development is without a doubt one of the most important processes that shape the life history of any organism, and changes in multiple physiological systems do not culminate with embryogenesis. Our study suggests that postnatal development of a marine mammal carnivore affects the way in which energetic resources are allocated to mount an inflammatory response to a given challenge. The immune system is a complex net of interacting mechanisms, which for a long time have been studied as isolated components. There is still little information regarding the synergism or antagonism that can occur among multiple immune system components. We have provided evidence that underlying immune activity might negatively affect the way in which an organism responds to a second immune challenge. Our study underscores the importance of taking into account the developmental stage of an organism, as well as its health status, when conducting ecoimmunological studies.

## Supplementary Material

We have blended all electronic supplementary material in a single file that contains three supplementary figures and one supplementary table.

## Data accessibility

The data used for this study has been stored in Dryad, http://dx.doi.org/10.5061/dryad.7sh05.
