# Supplementary material for: Activation of an inflammatory response is context-dependent during early development of the California sea lion
Source: R Soc Open Sci. 2015 Apr 29;2(4):150108. doi: 10.1098/rsos.150108 (PMC4448862; doi:10.1098/rsos.150108)
Supplement: We have blended all electronic supplementary material in a single file that contains three supplementary figures and one supplementary table. [file rsos150108supp1.docx]

**
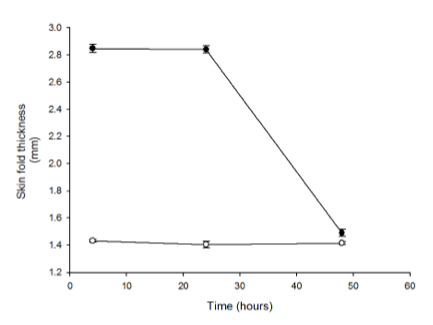
**

**Figure S1.** Average response to PHA injection of California sea lion pups at different times after exposure. Response was measured as the difference between the saline-induced change in median thickness of the left flipper and the PHA induced change in median thickness of the right flipper. Filled circles represent PHA-challenge flippers; empty circles represent saline-challenged flippers. Bars denote ± S.E.


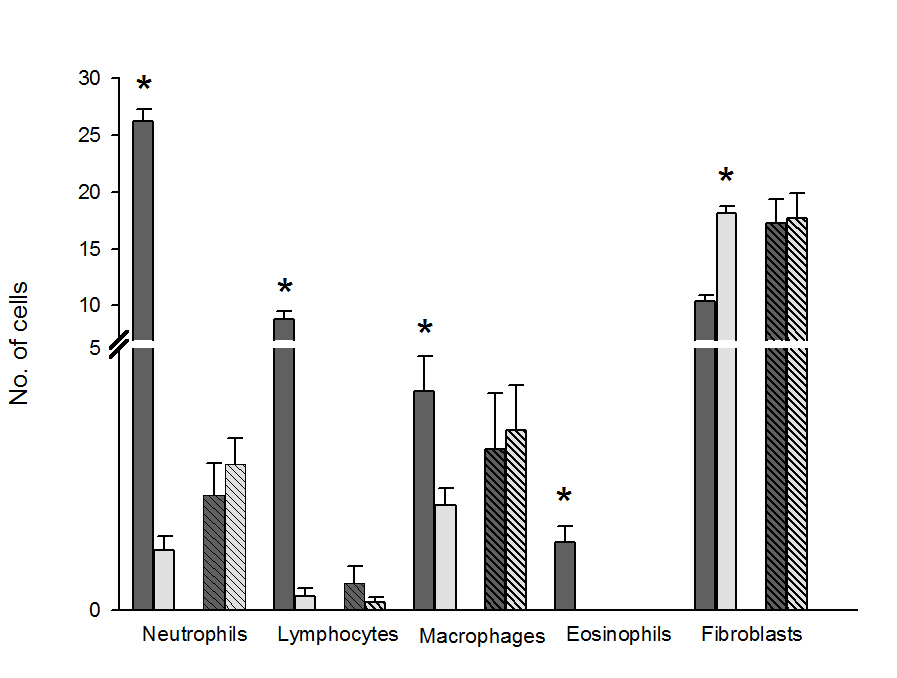


**Figure S2.** Mean cell counts of PHA-injected (dark grey) versus saline-injected (light grey) tissues of pups that showed significant swelling (no stripes) or no swelling at all (stripes). Asterisks denote significant differences (P<0.05).


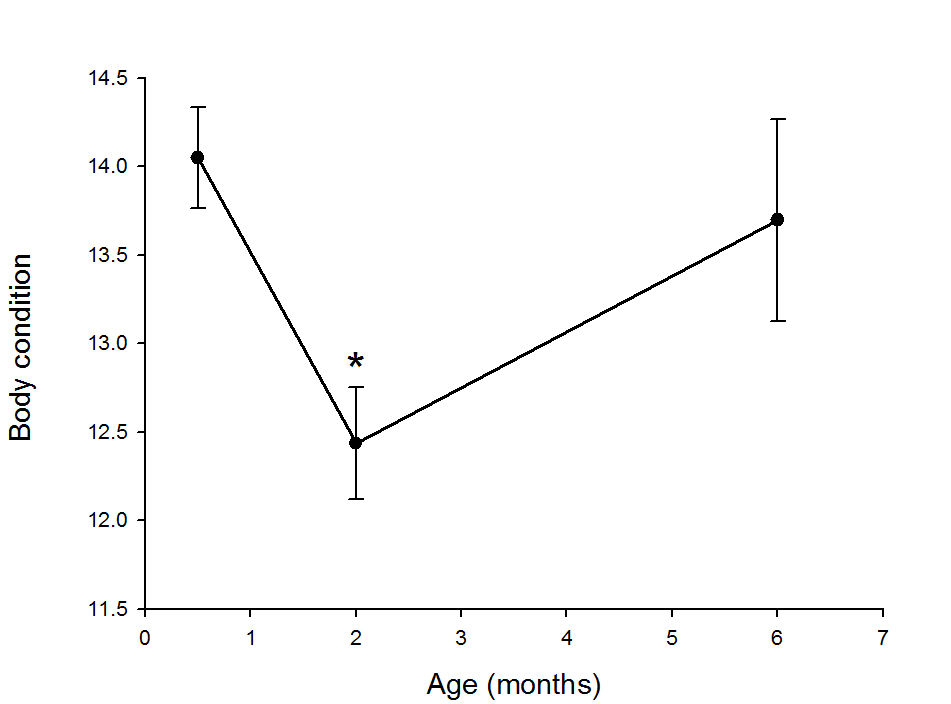


**Figure S3.** California sea lion pup body condition at different age points. Body condition was estimated as scaled mass per unit length (Peig & Green 2009). Lower or upper 95% confidence intervals are shown. Asterisks represent significant differences.

**Table S1.** Generalized linear models analyzing the effect of changes in A) body condition (*SM_i_*), circulating neutrophils (NEU), total circulating white blood cells (WBC), B) neutrophil to lymphocyte ratio (NLR) and their interactions on changes in skin fold thickness (Swelling) of neonate California sea lion pups.

1. Model: Swelling ~ $SM_{i}$* NEU*WBC, family=gaussian

| Variable | Slope | | F | P(>F) |
| --- | --- | --- | --- | --- |
| ${SM}_{i}$ | - | 0.4911 | | 0.3492 |
| NEU | - | 0.2389 | | 0.6849 |
| WBC | + | 0.4597 | | 0.4660 |
| ${SM}_{i}$:NEU | + | 2.3264 | | 0.1942 |
| ${SM}_{i}$:WBC | + | 0.3309 | | 0.5803 |
| NEU:WBC | - | 0.7537 | | 0.2328 |
| ${SM}_{i}$:NEU:WBC | + | 0.0011 | | 0.9078 |

N = 23, AIC = -27.65

1. Model: Swelling ~ ${SM}_{i}$* NLR*WBC, family=gaussian

| Variable | Slope | | F | P(>F) |
| --- | --- | --- | --- | --- |
| ${SM}_{i}$ | + | 0.4911 | | 0.3246 |
| NLR | + | 0.0044 | | 0.5066 |
| WBC | - | 0.6153 | | 0.4331 |
| ${SM}_{i}$:NLR | + | 1.1077 | | 0.3298 |
| ${SM}_{i}$:WBC | + | 0.5370 | | 0.5796 |
| NLR:WBC | - | 1.9739 | | 0.0785 |
| ${SM}_{i}$:NLR:WBC | + | 0.1094 | | 0.6530 |

N = 23, AIC = -25.23
